# Occupational risk factors for multiple sclerosis: a systematic review with meta-analysis

**DOI:** 10.3389/fpubh.2023.1285103

**Published:** 2023-11-16

**Authors:** Bruno Kusznir Vitturi, Alfredo Montecucco, Alborz Rahmani, Guglielmo Dini, Paolo Durando

**Affiliations:** ^1^Department of Health Sciences, University of Genoa, Genoa, Italy; ^2^Occupational Medicine Unit, IRCCS Ospedale Policlinico San Martino, Genoa, Italy

**Keywords:** multiple sclerosis, occupational, risk factors, environmental, demyelinating, epidemiology, etiology

## Abstract

**Objective:**

We decided to conduct the first systematic review with meta-analysis to provide the highest level of up-to-date evidence on the occupational risk factors for Multiple Sclerosis.

**Methods:**

A systematic, comprehensive literature search was performed in four electronic academic databases. We included any case-control study that enrolled working-age subjects and compared the proportion of MS cases with controls who were not exposed to an occupational risk factor. The primary outcome was the occurrence of MS. The quality assessment was performed with the Critical Appraisal Checklist for Case Control Studies, developed, and validated by the Joanna Briggs Institute. All the selection process was also carried out by two independent and previously trained researchers.

**Results:**

Overall, the total sample included 19,004 people with MS and 4,164,162 controls. Agricultural workers (OR = 1.44, 95% CI 1.13–1.83), offshore workers (OR = 3.56, 95% CI 2.74–4.61), and hairdressers (OR = 8.25, 95% CI 1.02–66.52) were associated with a higher probability of being diagnosed with MS. In parallel, workers exposed to toxic fumes from oil wells (OR = 16.80, 95% CI 8.33–33.90), low-frequency magnetic fields (OR = 1.71, 95% CI 1.03–2.72), and pesticides (OR = 3.17, 95% CI = 2.53–3.99) also had an increased likelihood of having MS.

**Conclusion:**

Our study has the potential to influence more assertive public policies. Nevertheless, future studies on how the occupational setting may contribute to the incidence of MS are highly recommended.

**Systematic review registration:**

The protocol was registered in the international prospective register of systematic reviews (PROSPERO– CRD42023443257).

## Introduction

Multiple Sclerosis (MS) is a chronic autoimmune disease that affects the central nervous system, causing demyelination and neurodegeneration (1, 2). Most individuals manifest the first symptoms between 20 and 40 years old. The disease affects women more than men, with a ratio of approximately 2:1 (3). The global prevalence is approximately 35.9 per 100,000 people and an estimated 2.8 million people worldwide are living with MS. Its incidence has been increasing globally every year since 2013, making it a subject of great public health concern (4). MS is one of the main causes of disability among young workers and it is associated with devastating socio-economic and occupational outcomes (5, 6).

Most chronic diseases still do not have a clear etiology. At the same time, environmental and occupational risk factors are increasingly related to diseases classified as ‘idiopathic’. Emerging evidence indicates that there is a complex and intimate ‘dialogue’ between intrinsic (genetic) and extrinsic (environmental and occupational risk factors) factors in the physiopathology of chronic and/or disabling diseases. Indeed, the Global Burden of Disease project considers up to 26 well-known environmental and occupational risk factors in its estimates (7). Moreover, the International Labour Organisation and the World Health Organisation (WHO) have estimated that 5–7% of deaths are attributed to occupational diseases and accidents at work (8). In 2012, the WHO estimated that 12.6 million global deaths, representing 23% (95% CI: 13–34%) of all deaths, were attributable to environmental factors (9). In contrast, the current knowledge about the occupational and environmental risk factors are far from being deemed satisfactory. At the same time, as the world’s population grows and life expectancy increases, the investment in primary prevention becomes an indisputable necessity. Research into occupational and environmental risk factors enables the development of effective public health strategies for the prevention of chronic diseases that are costly for health systems.

Several studies have been published linking environmental and occupational risk factors to neurological diseases other than MS. Current evidence suggests that exposure to specific metals, pesticides, solvents, and air pollution influence the incidence of dementia (10). Pesticides, herbicides, fungicides, and rural life have been considered risk factors for Parkinson’s disease as well (11). People who have worked more than 10 years in agriculture and have been exposed to thinners, paint removers, electromagnetic fields, fungicides, and specific metals have been associated with amyotrophic lateral sclerosis (12). Furthermore, a Swedish study revealed that there was an increased risk of epilepsy in certain occupations. Among male workers, waiters, staff in laundries, dry cleaners, office workers, construction workers, sales agents, and drivers were cited as having an increased risk. Among women, on the other hand, an increased risk of epilepsy was observed among cooks, stewards, administrators, and managers (13).

The scientific literature contains several articles that investigated the environmental risk factors related to multiple sclerosis. One of the environmental factors with the highest degree of evidence is sun exposure, which is believed to affect vitamin D levels, that are linked to the pathophysiology of MS (14). However, there is no doubt that this risk factor cannot be the only one associated with MS as the prevalence of the disease is also significant in countries with lower latitude and higher sun exposure. Recently, several studies have confirmed that most people with MS have evidence of previous Epstein-Barr virus (EBV) infection (15). If the literature is vast regarding the environmental risk factors for MS, no previous review has systematically addressed the potential occupational risk factors. As a result, the evidence on this topic is sparse and controversial. Therefore, we decided to conduct the first systematic review with meta-analysis in this field to provide the highest level of up-to-date evidence.

## Methods

In order to conduct this systematic review and meta-analysis, we followed the guidelines of the statement ‘Preferred Reporting Items for Systematic Reviews and Meta-analyses’ (PRISMA), the Joanna Briggs’ recommendations for systematic reviews of observational epidemiological studies reporting prevalence and cumulative incidence data and the guidelines of the meta-analysis of observational studies in epidemiology (MOOSE). The protocol was registered in the International Prospective Register of Systematic Reviews (PROSPERO – CRD42023443257). As this research did not involve the direct recruitment of subjects, local ethics committee approval was not required, and written consent forms were not necessary.

A systematic literature search was performed using four electronic academic databases – PubMed/MEDLINE, SciVerse ScienceDirect, Web of Science. The following search terms were used: (employ* OR occupation* OR work* OR job* OR environment* OR “exposure” OR “risk factors”) AND (“multiple sclerosis”). The search results were then exported and managed in Mendeley 1.19.8 (Elsevier, New York, United States). Two independent and adequately trained investigators carried out the selection of the studies (BV and AR), both of which were independent of the other’s decision and thus not influenced by each other. In case of discrepancies or conflicting opinions, a senior investigator (GD) was consulted to promote a discussion and reach a consensus. After removing duplicate entries, we made an initial selection of titles and abstracts to assess their potential relevance and remove those off-topic. The papers were then carefully read to determine their final eligibility. Inclusion criteria were framed according to the PECOS acronym. We included all original peer-reviewed articles that enrolled workers and compared the proportion of MS between those who were exposed and those who were not exposed to the occupational risk factor. We also included articles that investigated the incidence of MS considering specific job activities as the exposure. We excluded studies if it was not clear that exposure to the given risk factor was not predominantly work-related. There were no time limits and no language restrictions. We only included case-control studies or nested case-control studies, and therefore articles designed as reviews, clinical trials, conference abstracts, letters to the editor, expert opinions, commentaries, case reports, case series and editorials were excluded. Finally, multiple articles reporting the same outcome from the same study population were excluded as well.

The primary outcome was the occurrence of MS. When available, data on the first author, year of publication, country, sample size, mean age, gender, study design, job characteristics and occupational exposure history were extracted and tabulated in a Microsoft Excel spreadsheet. In the case of articles lacking essential data, we contacted the corresponding author to obtain more information by e-mail. Whenever our contact attempt failed, the study was excluded from the analysis. When a multicenter study reported results for each country, the information was treated as if it came from two different studies. All extracted data were double checked 1 month after the initial extraction to optimize reliability and minimize the risk of bias. The quality assessment was performed with the Critical Appraisal Checklist for Case Control Studies, developed and validated by the Joanna Briggs Institute. It comprises ten questions for which researchers can answer ‘yes’, ‘no’, ‘unclear’ or ‘not applicable (NA)’ in response to each item. The greater the number of ‘no’ or ‘unclear’ selected, the greater the risk of bias in each category and in each study. The critical appraisal was carried out considering the variables of interest in our review. This step was also carried out by two independent and previously trained researchers (BV and AM), always considering the opinion of a third researcher (GD) in case of discrepancy.

All quantitative data were pooled in a meta-analysis. We used the random-effects model based on the binomial distribution to calculate pooled estimates of the odds-ratio (OR) with respective confidence intervals (CI). The effect-sizes were calculated in log odd-ratios and were exponentiated to be reported. The influence of age and gender was assessed with meta-regression. Heterogeneity between the estimates was assessed by using the *I*^2^ statistic and visually inspecting the forest plot. An *I*^2^ greater than 75% was considered substantial heterogeneity. We investigated the existence of publication bias using Egger’s linear regression test and by visual inspection of the funnel plots. A *p*-value <0.05 was considered statistically significant. All the statistical analyses were performed with STATA/BE 17.0.

## Results

**Figure 1:**
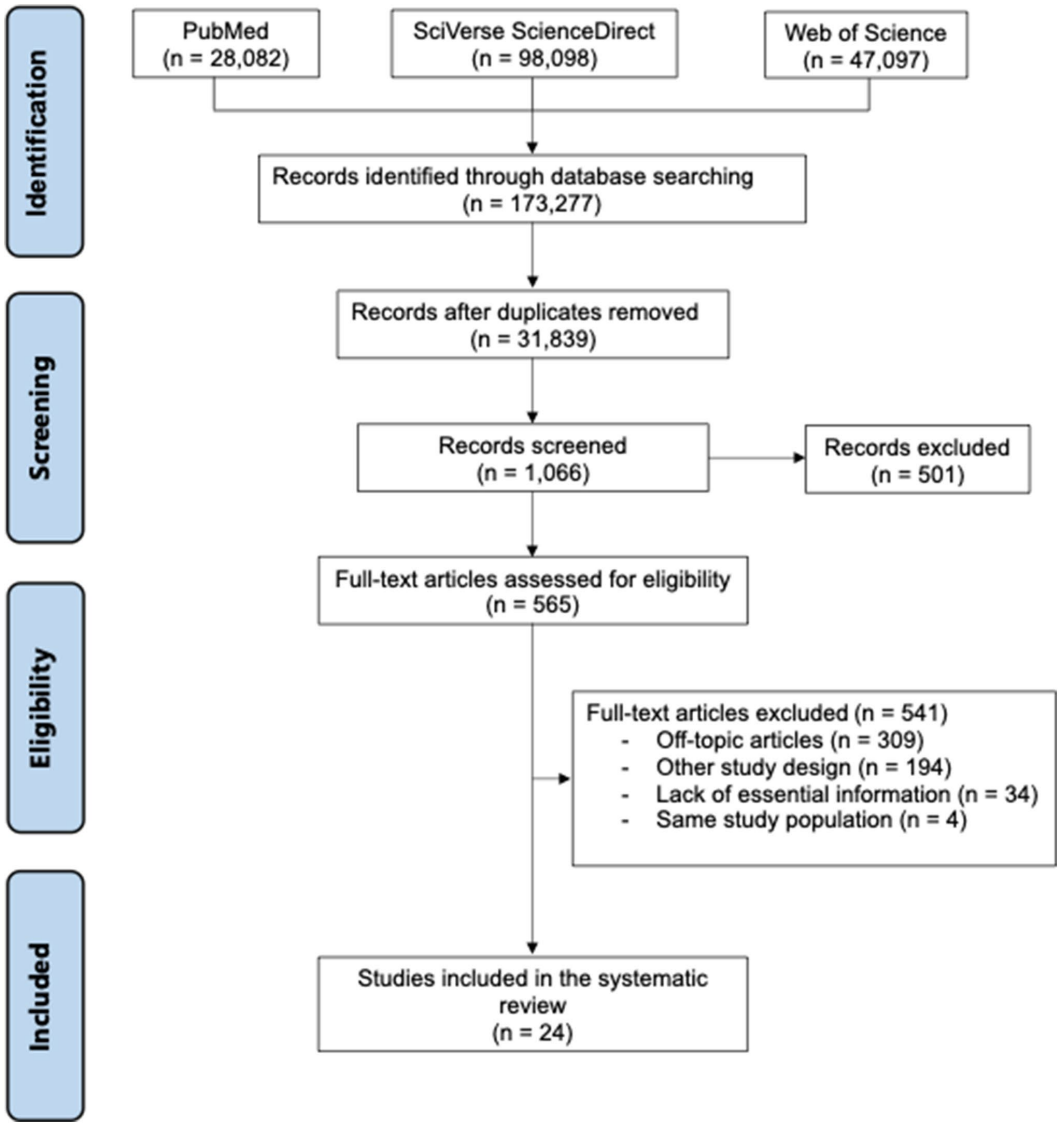
PRISMA flowchart.

One hundred and seventy-three thousand two hundred and seventy-seven articles matched the search terms. After excluding duplicates, 31,839 articles were examined according to the inclusion and exclusion criteria. Five hundred and one articles did not meet all the eligibility criteria, so 24 unique articles were included in our review. Figure 1 provides the PRISMA flow chart summarizing the entire article selection process. All studies were case-control studies. The studies were conducted in Australia (16), Denmark (17–20), France (21), Iran (22, 23), Italy (24, 25), Kuwait (26), Norway (27–29), Saudi Arabia (30), Spain (31), Sweden (32–37), and the United States of America (38, 39) (Table 1). The quality of the articles was variable, being the most common methodological flaw not considering or addressing the confounding factors properly (Supplementary Table S1).

**Table 1:** General description of the studies included in the review.

Abdollahpour et al.	2018	Iran	547	1,057	31,3	401 (73.3)	Joblessness Dismissal
Al Wutayd et al.	2018	Saudi Arabia	307	307	32,91	230 (74.9%)	Joblessness
Al-Afasy et al.	2012	Kuwait	101	202	33,85	56 (55.4%)	Joblessness Solvents Toxic fumes from oil wells
Amaducci et al.	1982	Italy	86	375,363	NA	NA	Shoe and leather workers
Anglen et al.	2015	USA	21	11,759	48,1	3 (14.3)	Mercury
Gronning et al.	2002	Norway	139	161	NA	NA	Solvents
Hedstrom et al.	2011	Sweden	1,343	2,900	33,4	73 (5.4)	Shift workers
Hedstrom et al.	2018	Sweden	1,601	2,947	NA	564 (35.2)	Solvents
Hedstrom et al.	2013	Sweden	1798	3,907	NA	1,301 (72.3)	Anaesthetic agents
Hedstrom et al.	2015	Sweden	2,337	4,904	NA	NA	Shift workers
Horwitz et al.	2013	Denmark	389	926,005	40,6	134 (34.4)	Construction workers Agricultural workers Trade, transport and industry workers
Landtblom et al.	1993	Sweden	91	348	NA	91 (100.0)	Solvents Radiation Animals
Landtblom et al.	2006	Sweden	907	41,756	NA	907 (100.0)	Solvents Animals
Magyari et al.	2014	Denmark	1,403	35,045	NA	939 (66.9)	Agricultural workers Healthcare workers Craftsman workers Chemical industry workers
Mortensen et al.	1997	Denmark	93	212,174	NA	0 (0.0)	Solvents
Motamed et al.	2014	Iran	65	85	30,7	43 (66.1)	Radiation
Oddone et al.	2013	Italy	334	1,336	NA	130 (38.9)	Shoe and leathers workers Construction workers Agricultural workers Healthcare workers Food industry workers
Papantoniou et al.	2019	USA	579	198,419	NA	579 (100.0)	Shift workers
Parron et al.	2011	Spain	682	1,832,287	NA	NA	Pesticides
Pedersen et al.	2007	Denmark	5,000	27,006	NA	0 (0.0)	Low-frequency magnetic fields
Riise et al.	2011	Norway	648	427,698	NA	236 (36.4)	Offshore workers
Riise et al.	2002	Norway	27	57,728	NA	NA	Solvents
Souberbielle et al.	2002	France	230	230	NA	NA	Healthcare workers Hairdressers Cleaners
Valery et al.	2013	Australia	276	538	NA	211 (46.4)	Agricultural workers Animals Soldiers

Overall, the total sample included 19,004 people with MS and 4,164,162 controls. The mean age ranged from 30.7 to 48.1 years and the percentage of women in the studies ranged from 0.0 to 100.0%. The oldest article was published in 1982, while the most recent article was published in 2019. Three articles evaluated the effect of joblessness as a risk factor for MS and one evaluated the effect of job dismissal. The articles examined occupational exposure to solvents, toxic fumes from oil wells, mercury, anesthetic agents, animals, radiation, pesticides, and low-frequency magnetic fields as potential risk factors for MS. The articles examined whether the following jobs could be linked to MS: shoemakers and leather workers, shift workers, construction workers, agricultural workers, trade, transport and industrial workers, offshore workers, healthcare workers, craftsmen, chemical industry workers, food industry workers, hairdressers, cleaners and soldiers.

**Figure 2:**
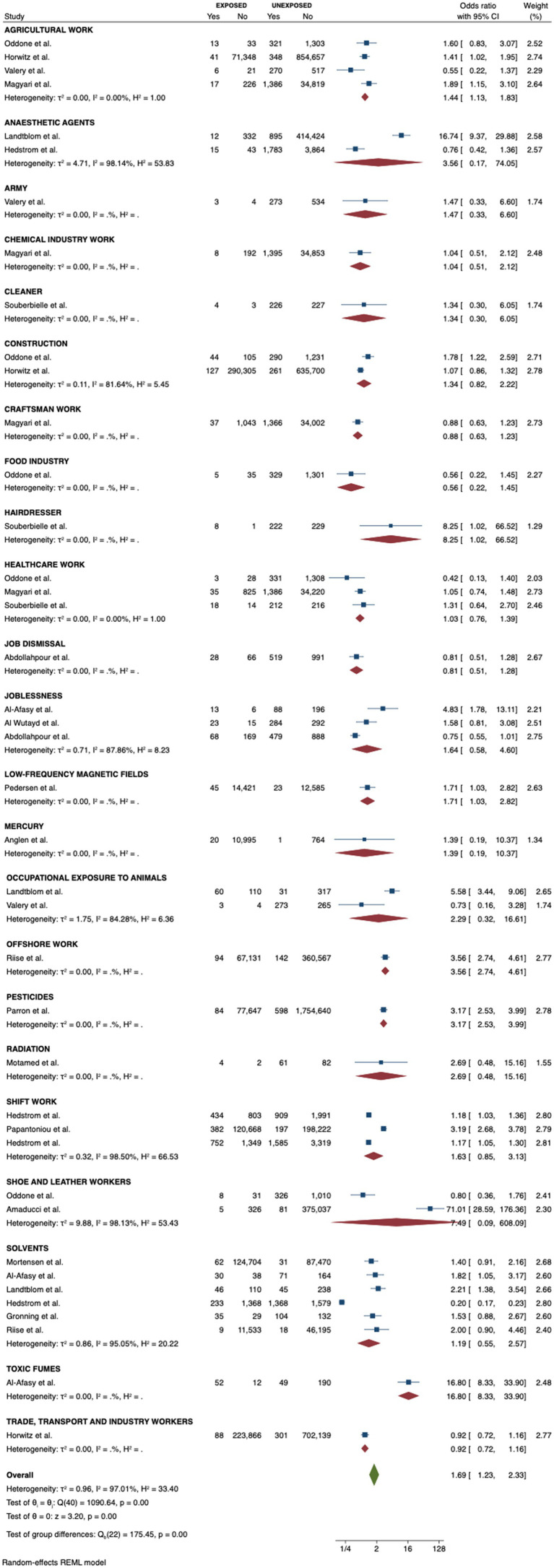
Forest plot of the meta-analysis results by subgroup.

**Figure 3:**
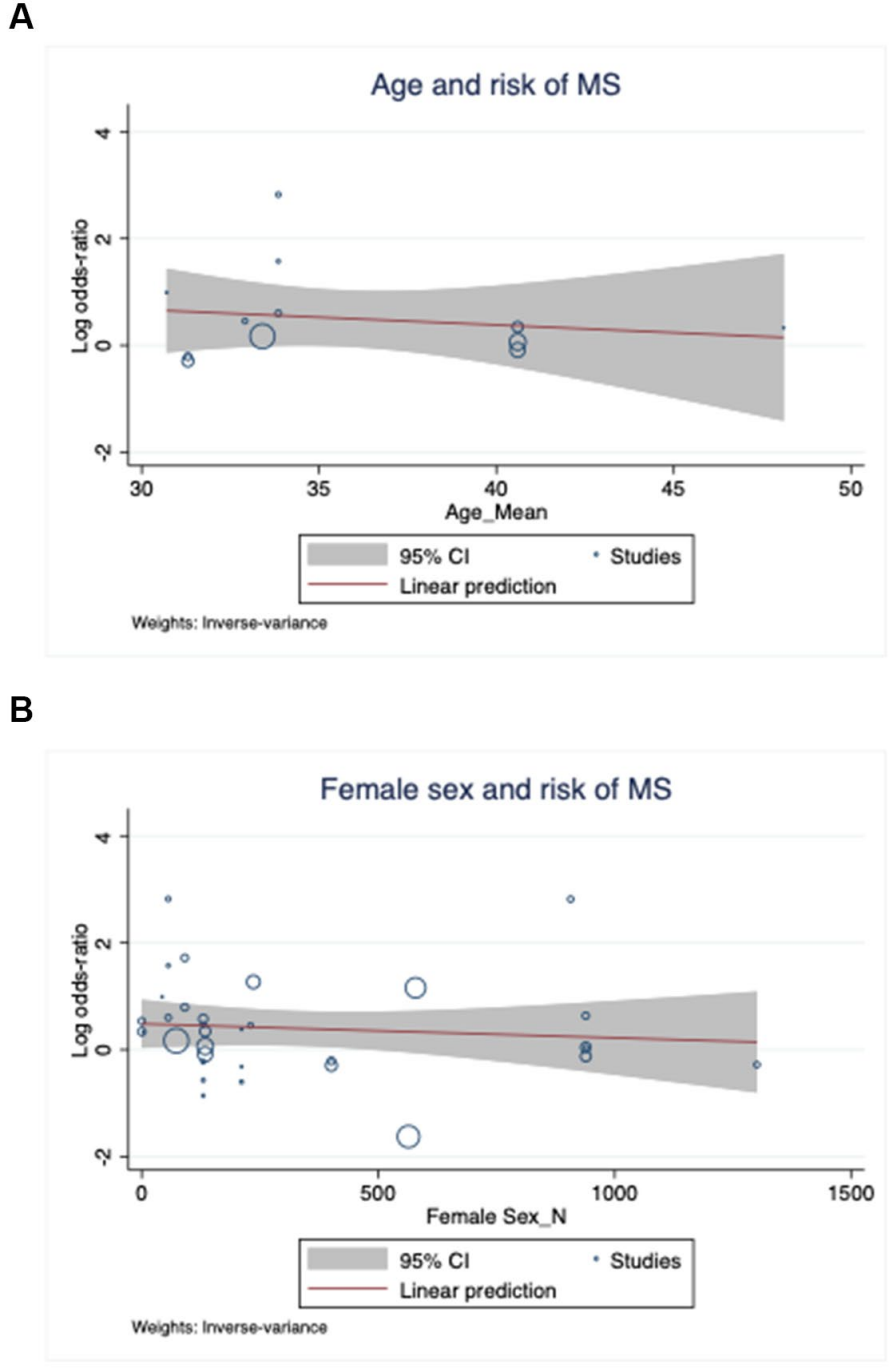
Meta-regression of the effect sizes considering age **(A)** and female sex **(B)** as moderators.

**Figure 4:**
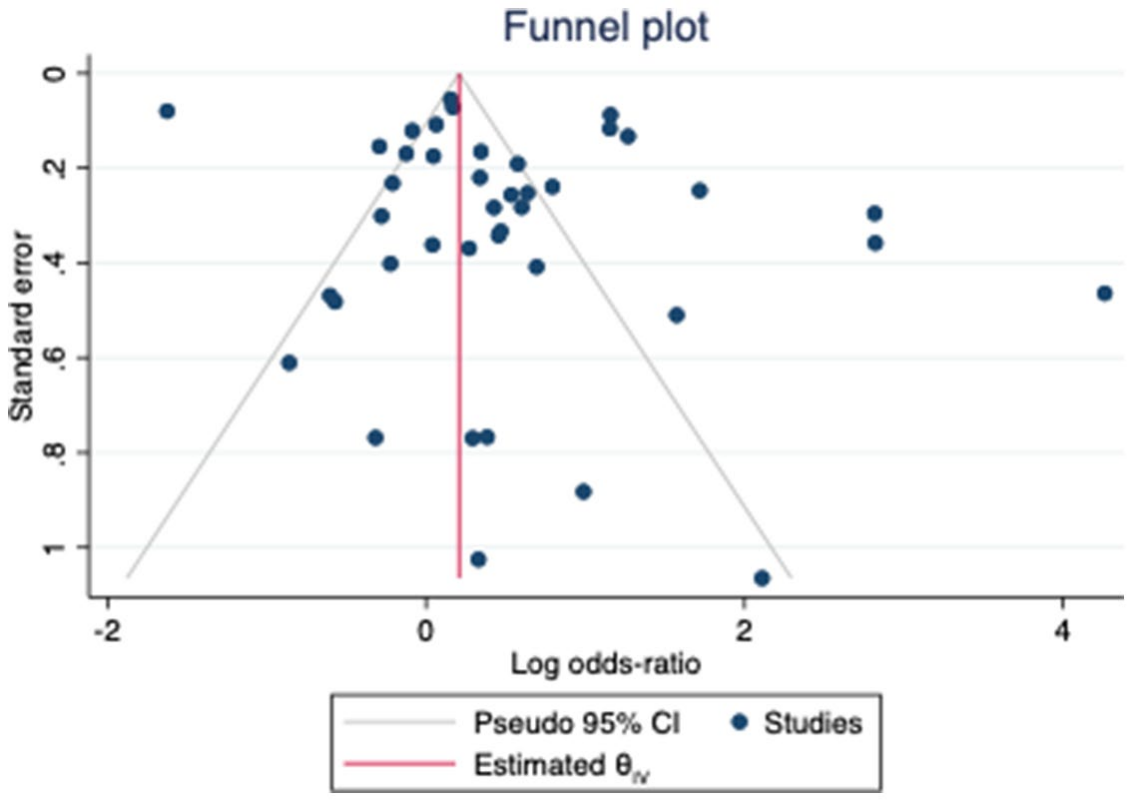
Funnel plot.

Agricultural workers (OR = 1.44, 95% CI 1.13–1.83), offshore workers (OR = 3.56, 95% CI 2.74–4.61), and hairdressers (OR = 8.25, 95% CI 1.02–66.52) were associated with a higher chance of being diagnosed with MS (Figure 2). In parallel, workers exposed to toxic fumes from oil wells (OR = 16.80, 95% CI 8.33–33.90), low-frequency magnetic fields (OR = 1.71, 95% CI 1.03–2.72), and pesticides (OR = 3.17, 95% CI = 2.53–3.99) also had an increased likelihood of having MS. Workers in the shoe and leather industry, shift workers, construction workers, trade, transport and industrial workers, healthcare workers, artisans, chemical industry workers, food industry workers, cleaners and military personnel did not have a different probability of developing MS compared to controls. Furthermore, occupational exposure to solvents, mercury, anesthetic agents, animals, and radiation were not risk factors for MS as well. The meta regression analysis showed no influence of age and sex in the effect sizes (Figure 3). The funnel plot and the egger’s test showed no publication bias (*p* = 0.40) (Figure 4).

## Discussion

This is the first comprehensive systematic review devoted to the investigation of possible occupational risk factors for MS. We reported all the occupational exposures ever studied on this subject. The literature is abundant on possible environmental risk factors that are not necessarily related to work, but our review showed that there is a significant lack of studies investigating work as a potential risk factor for MS. Studying the occupational setting is essential to protect the worker’s health. It also represents a unique opportunity to understand the role of the environment in the incidence of MS, as workers experience prolonged, continuous, and generally greater exposure to common environmental factors. For instance, numerous studies attest to the important role of sun exposure in the development of MS. However, the review revealed that there are no studies investigating the incidence of MS in workers naturally exposed to sunlight during the working day. In any case, the results need to be interpreted with caution since some studies did not present a satisfactory methodological quality.

The present study demonstrated that the chemical risk seems to be particularly important in relation to the incidence of MS. Indeed, the literature shows that several chemical substances can cause chronic neurological diseases and demyelination (40). On the other hand, knowledge about most toxic substances is still limited, especially considering their potential long-term effects on the central nervous system. In addition, many workers are exposed to chemical hazards that are often unknown or neglected by both the worker and the doctor in charge, which may underestimate the risk of MS due to an occupational chemical risk, as in the case of hairdressers. In other circumstances, workers may be exposed to a huge variety of potentially pathogenic substances that makes it difficult to identify a specific risk factor for MS, as in the case of workers exposed to toxic fumes from oil wells and offshore workers. Furthermore, this makes it difficult to draw specific conclusions about the pathophysiological mechanism of exposure to these various risk factors. In our study, the effect size for the subgroup of hairdressers was large, especially because it came from a single study, indicating that this finding deserves to be confirmed in more original articles.

Pesticides seem to be associated with MS. Unfortunately, papers do not specify the type of pesticide, neither their detailed chemical properties nor toxicity. Nevertheless, this group of chemical substances has already been associated with neurological diseases. Szepanowoski et al. believes that herbicides may trigger inflammation of peripheral nervous system that leads to demyelination (41). Arab et al. details the importance of the pesticide-induced neurotoxicity for several neurological diseases, including MS. The chronic inhalation of toxic substances and fumes diffused in the air also appears to be linked to MS (42). Offshore workers and workers exposed to toxic fumes had an increased probability of having MS. This finding is in line with previous studies that highlighted the air pollution as a well-known environmental risk factor for MS (43). Mohammedi et al. ratified this observation in a review that showed that inhalation of high concentration of toxic air pollutants can increase the risk of MS (44). Agricultural workers should be more vulnerable to MS. This finding may be supported by the exposure to chemical risk factors such as pesticides. In Norway, the prevalence of MS is higher in inland farming areas than in fishing villages (45, 46). Moreover, dairy operators seem to be more at risk for MS. The probable increased milk consumption has been listed as a possible hypothesis and a risk factor for MS (19). Another hypothesis come from the fact that these workers are exposed to various microorganisms (viruses, bacteria, fungi) and MS and demyelinating diseases have been increasingly linked to biological agents, especially viruses (47, 48). However, as our analysis has demonstrated, work-related biological risk is very little investigated by the scientific community.

Physical agents may also play a role in the pathogenesis of MS. The exposure to extremely low-frequency electromagnetic field was associated with increased incidence of MS. Preclinical studies showed that this kind of exposure may induce changes on the brain lipid profile (49). A narrative review shows that the electromagnetic field exposure may be associated with neuronal cell apoptosis and changes in the function of the nerve myelin and ion channels (50). Terzi et al. describe that Alzheimer’s Disease, Parkinson’s Disease, and Amyotrophic Lateral Sclerosis could be associated with the exposure to electromagnetic fields, that would be responsible for a pathological increase in the oxidative stress status (51).

The present review showed that the jobs associated with an increased probability of MS are mainly lower-skilled and low-income jobs, which may indicate that this group of workers may be more vulnerable to the disease. Another interpretation is that socio-economic factors are undeniably associated with the risk of MS. Social determinants of health are especially important in many neurological diseases (52). Kuhlmann et al. suggest the clinical course of MS may be better considered as a continuum, with contributions from concurrent pathophysiological processes that vary across individuals and over time (53). Dobson et al. state that social determinants of health are a multitude of individual factors and structural determinants that play a major role in all the stages of MS (54). In parallel, in our study, the meta-regression analysis showed an almost flat curve on the role of the female sex in the probability of MS, at the same time there is no doubt that women have a higher likelihood of being diagnosed with MS than men. This observation can be explained by the fact that men are more prevalent in the workforce in most professions considered to be at risk of MS.

Our study also has some limitations that must be acknowledged to allow an accurate interpretation of the results. Given the inherent methodology of a systematic review, it is not possible to exclude that there were some differences in the way the results were evaluated by the authors of each study, which could be responsible for some kind of methodological bias and significant heterogeneity. Unfortunately, most studies neither measured the degree of exposure to potential risk factors nor described them to exhaustion in the case of job characteristics and MS. There was an imbalance in the availability of literature across countries and thus our results may not be representative of some countries or regions. The influence of the country in terms of longitude and national income status could not be considered in our analysis. Moreover, it is known that patients with MS experience work difficulties from the earliest stages of the disease, including the pre-symptomatic phase, which can affect the likelihood of exposure (5, 55). Future studies should focus on the role of occupational risk factors in the pre-symptomatic/prodromal phase of the disease. Finally, there was limited evidence for some categories of occupational exposure and, therefore, there may be an issue with the external validity of the results of our review.

## Conclusion

MS is a chronic neurological disease whose cause has not been discovered yet. Environmental and occupational factors play an important role in the pathophysiology of many chronic diseases, including neurological diseases. This is the first comprehensive systematic review devoted to evaluating the occupational risk factors for MS. Our review identified that agricultural workers, offshore workers, and hairdressers were associated with an increased probability of being diagnosed with MS. In parallel, workers exposed to toxic oil well fumes, low-frequency magnetic fields, and pesticides also had an increased likelihood of having MS. The review highlights the need to pay more attention to specific job categories in terms of the likelihood of developing MS. The knowledge provided by this study may guide more assertive public policies for the primary prevention of MS and the protection of workers’ health. Future studies on how the occupational setting may contribute to the incidence of MS are also highly recommended.

## Data availability statement

The original contributions presented in the study are included in the article/Supplementary Material, further inquiries can be directed to the corresponding author.

## Author contributions

BV: Conceptualization, Data curation, Formal analysis, Investigation, Methodology, Project administration, Supervision, Writing – original draft, Writing – review & editing. AM: Data curation, Resources, Supervision, Writing – review & editing. AR: Conceptualization, Investigation, Methodology, Resources, Visualization, Writing – review & editing. GD: Conceptualization, Investigation, Methodology, Resources, Supervision, Writing – review & editing. PD: Conceptualization, Investigation, Methodology, Project administration, Resources, Supervision, Writing – review & editing.
