# Supplementary material for: Occupational risk factors for multiple sclerosis: a systematic review with meta-analysis
Source: Front Public Health. 2023 Nov 16;11:1285103. doi: 10.3389/fpubh.2023.1285103 (PMC10694508; doi:10.3389/fpubh.2023.1285103)
Supplement: Supplementary file 1 [file Table_1.docx]

| **Study** | **Q1** | **Q2** | **Q3** | **Q4** | **Q5** | **Q6** | **Q7** | **Q8** | **Q9** | **Q10** |
| --- | --- | --- | --- | --- | --- | --- | --- | --- | --- | --- |
| Mortensen et al. | UNCLEAR | UNCLEAR | YES | NO | UNCLEAR | NO | NO | UNCLEAR | UNCLEAR | YES |
| Pedersen et al. | UNCLEAR | UNCLEAR | YES | YES | YES | NO | NO | UNCLEAR | YES | YES |
| Anglen et al. | UNCLEAR | UNCLEAR | NO | YES | YES | YES | YES | NO | YES | YES |
| Motamed et al. | UNCLEAR | YES | NO | YES | YES | NO | NO | YES | UNCLEAR | YES |
| Al-Afasy et al. | YES | YES | NO | YES | YES | YES | YES | YES | YES | YES |
| Hedstrom et al. | YES | YES | YES | YES | YES | YES | YES | NO | YES | YES |
| Landtblom et al. | UNCLEAR | NO | YES | UNCLEAR | YES | YES | YES | YES | YES | YES |
| Oddone et al. | YES | YES | NO | YES | YES | NO | NO | YES | YES | YES |
| Horwitz et al. | UNCLEAR | UNCLEAR | YES | YES | YES | NO | NO | YES | YES | YES |
| Valery et al. | YES | YES | NO | NO | YES | YES | YES | NO | YES | YES |
| Al Wutayd et al. | YES | YES | YES | YES | YES | YES | YES | YES | UNCLEAR | YES |
| Riise et al. | YES | YES | YES | YES | YES | NO | NO | YES | YES | YES |
| Abdollahpour et al. | YES | YES | YES | YES | YES | YES | YES | YES | UNCLEAR | YES |
| Hedstrom et al. | YES | YES | YES | YES | YES | YES | YES | YES | YES | YES |
| Papantoniou et al. | UNCLEAR | UNCLEAR | YES | YES | YES | YES | YES | YES | YES | YES |
| Landtblom et al. | UNCLEAR | UNCLEAR | YES | YES | YES | NO | NO | NO | NO | YES |
| Magyari et al. | YES | YES | YES | YES | YES | NO | NO | YES | NO | YES |
| Hedstrom et al. | YES | YES | YES | YES | YES | YES | YES | YES | UNCLEAR | YES |
| Souberbielle et al. | YES | YES | YES | YES | YES | NO | NO | YES | NO | YES |
| Amaducci et al. | UNCLEAR | UNCLEAR | YES | NO | UNCLEAR | NO | NO | UNCLEAR | UNCLEAR | YES |
| Gronning et al. | UNCLEAR | UNCLEAR | NO | YES | YES | NO | NO | UNCLEAR | YES | YES |
| Riise et al. | NO | NO | UNCLEAR | YES | YES | NO | NO | NO | UNCLEAR | YES |
| Parron et al. | UNCLEAR | UNCLEAR | UNCLEAR | UNCLEAR | UNCLEAR | NO | NO | UNCLEAR | NO | YES |
| Hedstrom et al. | NO | NO | YES | YES | YES | YES | YES | YES | YES | YES |
